# Absence of mutations in *NR2E1 *and *SNX3 *in five patients with MMEP (microcephaly, microphthalmia, ectrodactyly, and prognathism) and related phenotypes

**DOI:** 10.1186/1471-2350-8-48

**Published:** 2007-07-26

**Authors:** Ravinesh A Kumar, David B Everman, Chad T Morgan, Anne Slavotinek, Charles E Schwartz, Elizabeth M Simpson

**Affiliations:** 1 Centre for Molecular Medicine and Therapeutics, Child & Family Research Institute, Department of Medical Genetics, University of British Columbia, 950 West 28^th ^Ave, Vancouver, V5Z 4H4, Canada; 2 Center for Molecular Studies, J.C. Self Research Institute, Greenwood Genetic Center. One Gregor Mendel Circle, Greenwood, South Carolina, 29646, USA; 3 Department of Pediatrics, Division of Medical Genetics, University of California, Box 0748, 533 Parnassus St., San Francisco, California, 94143-0748, USA

## Abstract

**Background:**

A disruption of sorting nexin 3 (*SNX3*) on 6q21 was previously reported in a patient with MMEP (microcephaly, microphthalmia, ectrodactyly, and prognathism) and t(6;13)(q21;q12) but no *SNX3 *mutations were identified in another sporadic case of MMEP, suggesting involvement of another gene. In this work, *SNX3 *was sequenced in three patients not previously studied for this gene. In addition, we test the hypothesis that mutations in the neighbouring gene *NR2E1 *may underlie MMEP and related phenotypes.

**Methods:**

Mutation screening was performed in five patients: the t(6;13)(q21;q12) MMEP patient, three additional patients with possible MMEP or a related phenotype, and one patient with oligodactyly, ulnar aplasia, and a t(6;7)(q21;q31.2) translocation. We used sequencing to exclude *SNX3 *coding mutations in three patients not previously studied for this gene. To test the hypothesis that mutations in *NR2E1 *may contribute to MMEP or related phenotypes, we sequenced the entire coding region, complete 5' and 3' untranslated regions, consensus splice-sites, and evolutionarily conserved regions including core and proximal promoter in all five patients. Two-hundred and fifty control subjects were genotyped for any candidate mutation.

**Results:**

We did not detect any synonymous nor nonsynonymous coding mutations of *NR2E1 *or *SNX3*. In one patient with possible MMEP, we identified a candidate regulatory mutation that has been reported previously in a patient with microcephaly but was not found in 250 control subjects examined here.

**Conclusion:**

Our results do not support involvement of coding mutations in *NR2E1 *or *SNX3 *in MMEP or related phenotypes; however, we cannot exclude the possibility that regulatory *NR2E1 *or *SNX3 *mutations or deletions at this locus may underlie abnormal human cortical development in some patients.

## Background

The MMEP phenotype and EEC syndrome represent syndromic forms of split-hand/foot malformation (SHFM), which occurs either as an isolated malformation or as a feature of many other conditions with overlapping clinical findings [1]. SHFM can be caused by chromosome rearrangements or single gene mutations and is a genetically heterogeneous malformation with multiple gene loci having been identified [1].

Sorting nexin 3 (*SNX3*) is ubiquitously expressed and belongs to the sorting nexin family, which is involved in intracellular protein trafficking [2]. Previously, *SNX3 *was found to be disrupted in its 3rd intron by a *de novo *balanced translocation t(6;13)(q21;q12) in a patient with MMEP (microcephaly, microphthalmia, ectrodactyly, and prognathism) and severe mental retardation [3]. Thus, *SNX3 *was initially proposed as a reasonable candidate for MMEP in this patient, who did not harbor any *SNX3 *mutations on the normal chromosome [3]. However, mutations involving *SNX3 *were not identified in a sporadic case with possible MMEP and normal karyotype [3,4]. Thus, mutations in a gene close to *SNX3 *may contribute to MMEP and related phenotypes.

**Figure 1:**
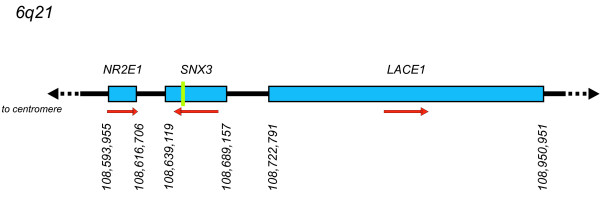
**Schematic drawing of 6q21 demonstrates relative positions of genes flanking SNX3**. Green bar indicates location of breakpoint within intron 3 of SNX3 in patient with t(6;13)(q21;q12) translocation [3]. Red arrows indicate direction of transcription. Genetic locations of transcriptional start and end sites are from RefSeq genomic assembly NC_000006.10.

Nuclear receptor 2E1 (*NR2E1*; previously, *TLX *[MIM 603849]) is the closest gene to *SNX3 *(Figure 1) and therefore represents a strong positional candidate that may contribute to the brain phenotype of MMEP. *NR2E1 *is also a strong functional candidate, given that mice deleted for *Nr2e1 *present with a complex MMEP-related phenotype that includes forebrain hypoplasia, eye abnormalities, and cognitive impairment [5-7], which is consistent with the brain and eye expression pattern of this gene [8,9]. Further evidence is supported by the possibility that the der(6) breakpoint in the t(6;13)(q21;q12) patient could have created a position effect that altered the expression of *NR2E1 *resulting in the MMEP phenotype (Figure 1) [3]. Such a hypothesis, however, is difficult to test in light of the predominantly brain-specific transcription of *NR2E1 *[8,10] and lack of suitable patient material.

We propose that a gene(s) near the 6q21 translocation may underlie MMEP and related phenotypes in some patients. Here, we test the hypothesis that patients with MMEP or a related phenotype may harbor mutations in *NR2E1 *and/or *SNX3*. To test this hypothesis, we sequenced the entire *NR2E1 *coding region, consensus splice-site regions, complete 5' and 3' untranslated regions and evolutionarily conserved elements including core and proximal promoter in one patient with MMEP, three patients with possible MMEP or related phenotypes, and one patient with oligodactyly, ulnar aplasia, and a t(6;7)(q21;q31.2) translocation [11]. We also sequenced the complete coding regions of *SNX3 *in three of five patients not previously examined for this gene.

## Methods

### Human subjects

Approval for this study was obtained from The University of British Columbia and Child & Family Research Institute. The research followed Canada's Tri-Council Statement on 'Ethical Conduct for Research Involving Humans'. Approval was also obtained through the Institutional Review Board of Self Regional Healthcare (Greenwood, SC). Consent from all patients was obtained for research purposes. Patients were ascertained and examined from five centers: The University of the Witwaterstrand, Johannesburg; The University of Cape Town, South Africa; Cedars-Sinai Medical Center, Los Angeles; Universita Cattolica, Rome; and The University of California, San Francisco. Patients were referred to the Greenwood Genetic Center for molecular research studies on split-hand/foot malformation. Controls were ascertained from the Greenwood Genetic Center.

All cases were sporadic and born to non-consanguineous parents. Patient 1 is the original patient with MMEP and t(6;13)(q21;q12) described previously by Viljoen and Smart [12]. This is a 44-year-old Caucasian female with severe mental retardation, congenital microphthalmia causing complete blindness, central cleft lip and palate, ectrodactyly with absence of toes 2–4 on both feet, finger-like thumbs, and a broad, prominent jaw.

Patients 2–4 had normal blood chromosome analyses and were included in this study on the basis of having clinical features that significantly overlapped those of the MMEP phenotype. Patient 2 was felt to have possible MMEP versus an unusual variant of the ectrodactyly-ectodermal dysplasia-clefting (EEC) syndrome and has not been described previously. She is a Hispanic female with congenital microcephaly, bilateral microphthalmia, colobomas of the right iris and retina, left chorioretinal coloboma, left lacrimal duct stenosis, unilateral cleft lip and palate, dysplastic ears, soft tissue syndactyly of fingers 3–4 on the right hand, ectrodactyly of the left foot with a hallux and two digital rays, unilateral mixed versus sensorineural hearing loss requiring a hearing aid, sparse scalp hair and eyebrows, and narrow, deep-set nails. She had a prolonged hospitalization after birth and required gastrostomy tube placement. She had no evidence of a *TP73L *(previously, *TP63*, P63) mutation, which is known to be involved in split hand/foot malformation [13].

Patients 3 and 4 were felt to have an unusual variant of the EEC syndrome. A brief summary of their clinical findings was reported previously [13]. Patient 3 is a 10-year-old Caucasian female with microcephaly, bilateral iris colobomas, microphthalmia with significant vision impairment, bilateral ectrodactyly of the hands and feet, unilateral cleft lip, and patchy alopecia of the scalp hair. She required gastrostomy feedings until age 9 due to ongoing problems with feeding, failure to thrive, and severe gastroesophageal reflux. A gastric emptying study revealed delayed emptying with a non-functioning section of the stomach and reverse peristalsis, which resolved with gastric Botox therapy. She has experienced significant dental problems due to ectodermal dysplasia. She attends a regular school program with visual assistance. Patient 4 is an African-American female seen at 3 months of age with congenital microcephaly, congenitally sealed eyelids with small to absent globes, ectrodactyly of the hands and feet, a notch in the upper lip resembling a mild midline cleft, absent scalp hair, underdeveloped eyelashes and eyebrows, underdeveloped nails, minor differences in ear shape, unilateral hearing loss, pelvic kidney, anteriorly placed anus, and tethered spinal cord. Both patients had no evidence of a *TP73L *mutation [13].

Patient 5 was included in the study on the basis of having a *de novo *chromosome translocation involving the 6q21 region (t(6;7)(q21;q31.2)) and congenital ulnar ray aplasia. His findings were described previously by Gurrieri et al. [11]. He is a Caucasian male evaluated in the newborn period with congenitally bowed radii, absent ulnae, absence of fingers 3–5 on the right hand, syndactyly of fingers 2–3 and absence of fingers 4–5 on the left hand, and a cyst of the septum pellucidum. He did not have microcephaly, ocular abnormalities, or other features of the MMEP phenotype. We cannot exclude the possibility that breakage at 7q31 may disrupt a gene(s) involved in MMEP.

### DNA amplification and sequencing

Sequencing was used to screen for *SNX3 *coding mutations in all 4 exons as previously described [3]. We sequenced genomic *NR2E1 *using 20 PCR amplicons that covered the coding regions (1,146 bp), complete 5' and 3' UTRs (1,973 bp), exon-flanking regions including consensus splice-sites (1,719 bp), and evolutionarily conserved regions including the core and proximal promoter (1,528 bp). Polymerase chain reactions (PCR) and sequencing were performed as previously described [14]. Sequences were visually inspected and scored blindly by at least two individuals using either Consed [15] or Sequencher (Gene Codes, Ann Arbor, MI). Every variant identified was confirmed by repeating the PCR and sequencing process. Confirmation of the g.21502T>C change in patient 2 and genotyping of unaffected parents and 250 controls without MMEP or related phenotypes was performed by restriction enzyme digestion of a 505-base pair PCR product using *Bsm*BI. Primers and PCR conditions were as previously reported for 3' UTRb [14]. The alteration created an additional *Bsm*BI site, producing bands of 505, 278, and 227 base pairs in the heterozygous state.

## Results and discussion

### Absence of SNX3 coding mutations in MMEP or related phenotypes

To exclude the role of coding mutations in *SNX3 *in MMEP or related phenotypes, we sequenced all four coding exons in all patients not previously studied for this gene. No mutations were detected. The absence of coding mutations in *SNX3 *supports the role of other loci, such as *NR2E1*, in these disorders. We cannot, however, exclude the role of regulatory *SNX3 *mutations given that promoter and UTRs were not examined.

### Absence of NR2E1 coding mutations in MMEP or related phenotypes

To determine whether patients with MMEP or related phenotypes harbor *NR2E1 *mutations, we sequenced the complete coding region, complete 5' and 3' UTR, consensus splice-sites, and evolutionarily conserved regions including the core and proximal promoter in all five patients. We generated approximately 33.5 kb of sequence data. We did not detect any synonymous nor nonsynonymous coding mutations.

**Figure 2:**
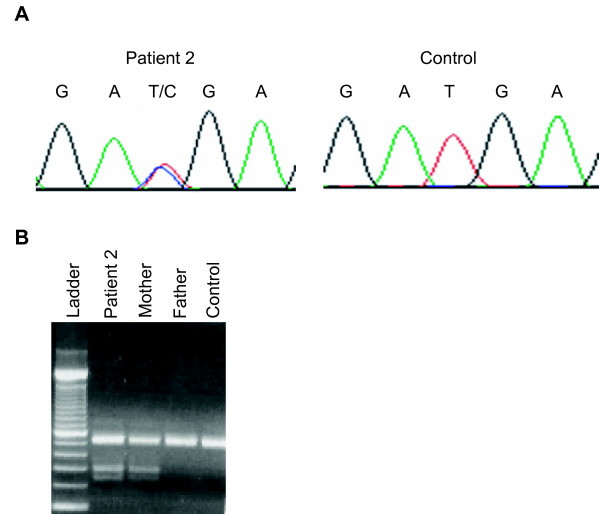
**Genotyping NR2E1 candidate regulatory mutation in patient 2 and family members**. A) DNA sequencing detects g.21502T>C in the 3' UTR of patient 2 but not in unrelated controls. B) Restriction digest detects g.21502T>C in the unaffected mother but not in the unaffected father nor in an unrelated control.

We identified one candidate regulatory mutation in the 3' UTR, g.21502T>C, in patient 2, that was not found in dbSNP (Build 127) [16]. We confirmed this variant by resequencing both strands of DNA. The variant was not predicted to alter binding of neural transcription factors [14]. We genotyped g.21502T>C in the unaffected parents and identified the candidate regulatory mutation in the mother but not the father (Figure 2). In addition, we did not find g.21502T>C in 500 chromosomes from approximately 250 controls without MMEP or related phenotypes. Interestingly, we previously reported the g.21502T>C candidate regulatory mutation (in addition to two other mutations) in a patient with microcephaly and the unaffected father but not in 344 control chromosomes nor in 188 ethnically-diverse chromosomes [14]. Thus, g.21502T>C has so far been identified only in families that present with microcephaly but not in 1032 control chromosomes from subjects that do not present with microcephaly.

One possibility is that the g.21502 T>C is an innocuous substitution that does not contribute to disease but rather represents a rare variant in the general population. An alternative interpretation is that heterozygous *NR2E1 *mutations may contribute to disease, which is supported by studies in mice heterozygous for *Nr2e1 *deletions that show premature cortical neurogenesis early in development [17], thereby suggesting dosage sensitivity for *NR2E1*. The presence of g.21502T>C in an unaffected parent may be due to incomplete penetrance of this variant. Alternatively, g.21502T>C may interact with another susceptibility variant arising *de novo *in the patient or inherited from the non-g.21502T>C-transmitting parent. Such a mechanism is supported by genetic studies in mice in which double heterozygous mutations at *Nr2e1 *and *Pax6 *are shown to enhance cortical phenotypes [18]. It is important to note that the clinical phenotypes of the patients in this study may be attributable to different genetic mechanisms affecting similar developmental pathways.

## Conclusion

MMEP and related phenotypes represent a spectrum of heterogeneous conditions for which multiple loci may be involved, including *NR2E1 *and *SNX3 *on Chromosome 6q21–22 [11,12]. The present study does not support involvement of *NR2E1 *or *SNX3 *coding mutations in MMEP or related phenotypes. However, we cannot exclude the possibility that regulatory *NR2E1 *or *SNX3 *mutations, such as g.21502T>C of *NR2E1*, may underlie abnormal human cortical development in some families. In addition, we cannot exclude the possibility that deletions at *NR2E1 *or *SNX3 *may underlie MMEP, given that sequencing is unable to distinguish between homozygosity across loci versus large deletions. The lack of obvious mutations in *NR2E1 *and *SNX3 *contribute to the genetic complexity underlying this heterogeneous syndrome. Follow-up studies of other positional candidates such as *LACE1*, would be a next logical undertaking.

## Competing interests

The author(s) declare that they have no competing interests.

## Authors' contributions

RAK conducted the *NR2E1 *molecular genetic studies, performed data analysis, and wrote the manuscript. DBE, AS, and CES collected families and provided DNA. CTM carried out the *SNX3 *sequencing and contributed to *NR2E1 *genotyping. EMS initiated the study and finalized the analyses as well as the paper. All authors read and approved the final manuscript.

## Pre-publication history

The pre-publication history for this paper can be accessed here:


